# Zebrafish as an Animal Model in Cannabinoid Research

**DOI:** 10.3390/ijms241310455

**Published:** 2023-06-21

**Authors:** Joanna Lachowicz, Aleksandra Szopa, Katarzyna Ignatiuk, Katarzyna Świąder, Anna Serefko

**Affiliations:** 1 Department of Clinical Pharmacy and Pharmaceutical Care, Medical University of Lublin, 1 Chodźki Street, 20-093 Lublin, Poland; joanna.lachowicz@umlub.pl (J.L.); anna.serefko@umlub.pl (A.S.); 2 Scientific Circle, Department of Clincal Pharmacy and Pharmaceutical Care, Medical University of Lublin, 1 Chodźki Street, 20-093 Lublin, Poland; 57645@student.umlub.pl; 3 Chair and Department of Applied and Social Pharmacy, Medical University of Lublin, 1 Chodźki Street, 20-093 Lublin, Poland; katarzyna.swiader@umlub.pl

**Keywords:** cannabinoids, endocannabinoids system, zebrafish, *Danio rerio*, animal model

## Abstract

Cannabinoids are active substances present in plants of the *Cannabis* genus. Both the Food and Drug Administration (FDA) and European Medicines Agency (EMA) have approved several medicinal products containing natural cannabinoids or their synthetic derivatives for the treatment of drug-resistant epilepsy, nausea and vomiting associated with cancer chemotherapy, anorexia in AIDS patients, and the alleviation of symptoms in patients with multiple sclerosis. In fact, cannabinoids constitute a broad group of molecules with a possible therapeutic potential that could be used in the management of much more diseases than mentioned above; therefore, multiple preclinical and clinical studies on cannabinoids have been carried out in recent years. *Danio rerio* (zebrafish) is an animal model that has gained more attention lately due to its numerous advantages, including easy and fast reproduction, the significant similarity of the zebrafish genome to the human one, simplicity of genetic modifications, and body transparency during the early stages of development. A number of studies have confirmed the usefulness of this model in toxicological research, experiments related to the impact of early life exposure to xenobiotics, modeling various diseases, and screening tests to detect active substances with promising biological activity. The present paper focuses on the current knowledge of the endocannabinoid system in the zebrafish model, and it summarizes the results and observations from studies investigating the pharmacological effects of natural and synthetic cannabinoids that were carried out in *Danio rerio*. The presented data support the notion that the zebrafish model is a suitable animal model for use in cannabinoid research.

## 1. Introduction

The history of the medical use of plants belonging to the *Cannabis* genus dates back to 2700 BC [1]. 

Historical evidence indicates that *Cannabis* was applied as a painkiller, anesthetic, antispastic agent, and an anxiety-reducing herb. However, their hallucinogenic effects after ingestion in large amounts were known even at those times. Data from ancient Egypt say that hemp was administered orally, rectally, vaginally, to the eye, and on the skin [2]. 

Until today, more than 560 compounds have been identified in hemp [3], including the main component Δ^9^-tetrahydrocannabinol (Δ^9^-THC), which has psychotropic properties. Additionally, hemp contains nonpsychoactive cannabinoids, such as cannabinol (CBN), cannabitriol (CBT), cannabinodiol (CBND), and the most promising compound due to its high potential for use in pharmacotherapy—cannabidiol (CBD). In the human body, both Δ^9^-THC and CBD are metabolized in the liver via the cytochrome P450 (CYP 450) system, and they undergo an extensive first-pass effect when taken orally [4,5]. Δ^9^-THC and CBD are highly lipophilic, and have poor oral bioavailability (up to 6%) [6]. The most common way of administering marijuana, especially for recreational purposes, is inhalation. Whether marijuana is administered via smoking or using a vaporizer, bioavailability after this way of administration is much better than after the oral form and reaches up to 10 to 35%. Furthermore, the first-pass effect is significantly reduced [7]. Similarly, in comparison to the oral form, better absorption of active ingredients present in *Cannabis* is observed when using the so-called oromucosal form (this form is available in the pharmaceutical market, and it contains the *Cannabis* extract) and when using transdermal patches (in experimental studies) [5,8]. Marijuana-active compounds are excreted mainly in the feces (at more than 65%), and about 20% are found in urine [9,10]. Generally, cannabinoids form a broad group of biologically active substances. Their mechanism of action is based on interactions with cannabinoid (CB) receptors. These compounds, according to their origin, have been divided into three categories: endocannabinoids, phytocannabinoids, and synthetic cannabinoids [11]. Recently, allosteric modulators of the CB1 receptors and compounds acting on enzymes participating in endocannabinoid synthesis or degradation have gained particular attention as potential therapeutic targets (Table 1) [12]. Endocannabinoids, anandamide (AEA), and 2-arachidonoylglycerol (2-AG) are endogenous ligands of CB receptors. They are synthesized “on demand” by postsynaptic cells without intermediate storage in synaptic vesicles, and they are released into the synaptic cleft, where they act as backward synaptic transmitters by binding to CB receptors situated on presynaptic terminals of neurons [13,14,15,16,17,18,19]. Some synthetic cannabinoids (i.e., nabilone, dronabinol) are already used in the treatment of drug-resistant epilepsy, vomiting, nausea, anorexia, and in multiple sclerosis, but it is also the fastest-growing group of illicit compounds with an alarming side effect profile that is often difficult to explain by the established molecular mechanisms of cannabinoid pharmacology. Furthermore, they are used as research tools in experimental studies. Importantly, synthetic cannabinoids should not be confused with synthetic phytocannabinoids, i.e., THC or CBD obtained by chemical synthesis.

CB receptors belong to G protein-coupled metabotropic receptors. So far, two types of CB receptors have been described: CB1 and CB2 receptors [11]. The binding of endocannabinoids to CB1 receptors leads to an inhibition of adenylyl cyclase [18,20], the opening of potassium channels, the closing of calcium channels, and the stimulation of protein kinases [21], whereas the interaction of endocannabinoids with CB2 receptors results in neuromodulatory effects [22]. The highest concentration of CB1 receptors has been detected in the hippocampus, cerebral cortex, cerebellum, amygdala, and others. Their activation leads to an inhibition of the release of various neurotransmitters (i.e., glutamate, gamma-amino butyric acid, dopamine, acetylcholine), and thus, they are responsible for the central effects of cannabinoids, including the influence on appetite, memory processes, learning, sleep, or emotional states [23]. Activation or inhibition of CB1 receptors in the central nervous system can alleviate the symptoms of various diseases, i.e., anxiety, depression, obesity, multiple sclerosis, Parkinson’s disease, Alzheimer’s disease, Huntington’s disease, and others [18,24,25]. Apart from the central nervous system, CB1 receptors are localized in various peripheral tissues, i.e., they are present in the gastrointestinal tract, heart, lungs, liver, and kidneys. In turn, CB2 receptors can also be found in the central nervous system, but they are mainly situated on immune cells, particularly on B lymphocytes, macrophages, monocytes, and natural killer cells. Due to their location, CB2 receptors have an influence on the immune system, and they participate in inflammatory processes [23]. It was demonstrated that Δ^9^-THC has higher (about 10 times) affinity to CB1 and CB2 receptors than CBD [26,27].

**Table 1:** Compounds affecting the endocannabinoid system.

Type of Compounds	Description
Endocannabinoids	Compounds naturally occurring in living organisms, arachidonic acid derivatives Examples: nandamide (AEA), 2-arachidonoylglycerol (2-AG) Significance: influence on emotions, metabolism, appetite, memory and learning processes, and psychomotor activity [28]
Phytocannabinoids	Compounds of herbal origin Examples: Δ^9^-tetrahydrocannabinol (Δ^9^-THC), cannabidiol (CBD) Significance: psychoactive effects (Δ^9^-THC), therapeutic potential (CBD) Side effects (particularly after high doses of Δ^9^-THC): palpitations, paranoia, anxiety, nausea, vomiting, confusion, coordination disturbances, and seizures [29]
Synthetic cannabinoids	Synthetic compounds Examples: nabilone, dronabinol Significance: used in experimental pharmacology, and used in the treatment of drug-resistant epilepsy, vomiting and nausea during chemotherapy, anorexia in AIDS patients, used as an adjunctive treatment in multiple sclerosis Side effects: similar to side effects observed after high doses of THC, mentioned above [30]
Allosteric modulators	Small molecules that allosterically modulate the CB1 receptor Examples: Org27569, PSNCBAM-1, ZCZ011, pepcan-12, lipoxin A_4_, pregnenolone Significance: better receptor subtype selectivity than cannabinoids, selectively towards tissues where the endogenous ligands are present and function, “ceiling” action preventing drug overdose [14]
Molecules degrading the endogenous cannabinoid ligands	Inhibitors of fatty acid amide hydrolase (FAAH) Examples: substrate-derived inhibitors (trifluoromethyl ketone analog of oleamide, MAFP), α-ketoheterocycle inhibitors, carbamate inhibitors, urea inhibitors, aryl boronic acids Significance: potential use in the treatment of pain, inflammation, multiple sclerosis, Parkinson’s disease, Alzheimer’s disease, insomnia, anxiety, depression, hypertension, cancer, and inflammatory bowel disease [12] Inhibitors of monoacylglycerol lipase (MAGL) Examples: maleimide derivatives, disulfides (e.g., disulfiram), carbamates (URB602, JZL184, KML29, ABX-1431), ureas, arylthoamides, tetrahydrolipstatin-based inhibitors, isothiazolines (e.g., octhilinone), natural terpenoids (e.g., pristimerin, euphol), amide-based derivatives Significance: potential use in the treatment of pain, anxiety, inflammation, cancer, and metabolic disorders [13]

### 1.1. Potential Use of Cannabinoids in Medicine

The interest in cannabinoids and their potential use in medicine is still growing. Due to their broad central and peripheral activity, scientists and the medical world have high hopes in relation to compounds acting on CB1 and CB2 receptors. One of the “urgent” health problems in which cannabinoids could be beneficial is drug-resistant epilepsy, particularly in children. According to the reviews by Rosenberg et al. [31] and by Gaston and Friedman [6], most studies carried out in rodents demonstrated an anticonvulsant activity of Δ^9^-THC and CBD, and their synergistic effects with classic anti-epileptic drugs, i.e., phenytoin and phenobarbital. The authors admit that there are several reports showing inconclusive results, but these divergences could be due to individual differences in the expression of CB receptors. Furthermore, cannabinoids have a proven anticancer effect, which is attributed to their influence on autophagocytosis and apoptosis. Outcomes of multiple clinical studies indicate a high potential of cannabinoids not only in the alleviation of side effects associated with cancer therapy but also in the application of these agents as adjunctive drugs acting synergistically with chemotherapeutics. In addition, in preclinical experiments, cannabinoids increased the survival rate of mice with induced cancer [32]. Researchers also draw attention to the possible neuroprotective effect of cannabinoids, which could be observed as a result of the complex mechanism involving not only the direct activity towards CB1 and CB2 receptors but also the indirect activity towards serotonin receptors and other processes, including those related to the reduction of oxidative stress [33]. There are reports indicating that in some diseases, such as migraines, irritable bowel syndrome, multiple sclerosis, or Parkinson’s disease, the functioning of the endocannabinoid system may be weakened; thus, in these health problems, the use of phytocannabinoids could be beneficial [34]. 

Currently, natural cannabinoids and their synthetic derivatives can be found in several medicinal products available in the pharmaceutical market in the United States and Europe. Their therapeutic indications approved by the Food and Drug Administration (FDA) and/or European Medicines Agency (EMA) are presented in Table 2.

### 1.2. Preclinical Research of Cannabinoids

Preclinical research is needed in order to introduce more cannabinoid-based products into the pharmaceutical market. Due to a variety of new potential indications and a large database of synthetic cannabinoids that could be tested, it is necessary to search for novel, more effective methods to carry out these studies. Mice and rats are well-recognized laboratory animals for testing cannabinoids. However, experiments with their use have several disadvantages; they are expensive, time-consuming, require quite a lot of space in an animal facility, etc. [35,36]. In order to screen a number of agents in a relatively short time and to reduce research costs, scientists increasingly use the zebrafish model in their labs. *Danio rerio* (zebrafish) is a small tropical freshwater fish that derives from the river Ganges and its tributaries in northern India. *Danio rerio* is evolutionarily closer to humans than invertebrate models, but it is more distant from humans than rodent models. It has advantages similar to invertebrate models, such as high fecundity, which is associated with easy access to a large number of subjects for research, small size, rapid development, and a short life cycle. Administration of tested agents (particularly small compounds) to zebrafish is easy since *Danio rerio* can absorb them from the surrounding medium/water via the skin. Furthermore, the zebrafish genome is fully sequenced and is significantly similar to the human one. The zebrafish genome is easily manipulated with relatively simple techniques. In addition, particularly when thinking about the assessment of an influence of a given substance on the fetus, an important feature of *Danio rerio* is the external (i.e., ex-utero) development and transparency of the embryo/larvae during the early stages of its development. It enables easy observation of drug effects under a microscope [18,37]. The zebrafish model is also in line with the 3R principles (Replacement, Reduction, and Refinement) [38]. Another important thing in relation to the present work is the fact that zebrafish have a well-described endocannabinoid system. As a consequence, it makes *Danio rerio* an ideal model also for testing cannabinoids.

The present paper focuses on the current knowledge of the endocannabinoid system in the zebrafish model and summarizes the results and observations from studies investigating the pharmacological effects of natural and synthetic cannabinoids that were carried out in *Danio rerio*.

## 2. The Endocannabinoid System in *Danio rerio*

The endocannabinoid system is composed of CB1 and CB2 receptors, endocannabinoids, (AEA and 2-AG), and enzymes responsible for their biosynthesis and degradation, including diacylglycerol lipase, N-arachidonoyl phosphatidyl ethanol-preferring phospholipase D, glycerophosphodiesterase 1, α,β-hydrolase domain–containing 4, fatty acid amide hydrolase, monoglyceride lipase, and others [39]. It has been demonstrated that the endocannabinoid system is highly conserved between *Danio rerio* and mammals. Molecular studies of CB1 and CB2 in zebrafish revealed a respective 66–75% and 39% amino acid similarity with their mammalian (including humans) counterparts [40,41]. The signaling dependent on CB receptors plays a significant role during the early development of zebrafish. It affects the hatching process, survival, mobility, development of motor neurons, heart rate, and responses to auditory and mechanical stimuli [42,43,44,45]. Inhibition of CB receptors 48 h before hatching leads to locomotor and morphological deficits [45,46].

In lysates from the whole body of zebrafish, *CB1* mRNA is detected as early as ca. 10 h postfertilization (hpf), whereas the expression of the *CB1* gene in the central nervous system is detected after 1-day postfertilization (dpf) in the preoptic area, and during the next day also in the telencephalon, hypothalamus, tegmentum, and anterior hindbrain. The expression pattern of the *CB1* gene in the central nervous system of *Danio rerio* shares homologies with the one observed in mammals [25]. According to the literature data [47], a high density of CB1 receptors in zebrafish can be found in the medial pallium of the dorsal telencephalon and the lateral pallium. The medial pallium bears signs of a functional homology to the mammalian amygdala, whereas the lateral pallium can be treated as the mammalian hippocampus. Both the amygdala and hippocampus in mammals also have a high density of CB1 receptors. CB1 receptors in *Danio rerio* can also be found in peripheral tissues, including the liver and ovary. Knockdown of the *CB1* gene in zebrafish resulted in disturbed axonal growth and the fasciculation of reticulospinal neurons [48]. There is still no precise data on the expression of CB2 receptors in zebrafish, but *CB2* mRNA has been detected both in the brain and in the periphery, including the spleen (i.e., high expression in the spleen suggests the modulatory effects of CB2 receptors on immune functions), heart, muscle, intestine, pituitary, gills, and retina [25,49]. According to Oltrabella et al. [18], the expression of CB2 receptors starts as early as around 1 hpf. Importantly, it seems that *Danio rerio* express a single form of CB1 receptor [41], and two forms of CB2 receptors [49]. Therefore, during the early stages of development (until the gastrulation phase), levels of CB2 receptors are higher than the ones detected for CB1 receptors, suggesting their more significant role. However, by the hatching process, the expression of CB1 receptors greatly increases which is accompanied by a decrease in the expression of CB2 receptors. Consequently, it suggests that CB1 receptors could play a more important role during later stages of development [18]. Moreover, Acevedo-Canabal and colleagues [50] demonstrated that the CB2 loss-of-function stable mutant zebrafish line (CB2-KO) presented swimming disturbances and anxiety-like behavior. In compliance with these observations, it seems that the signaling dependent on CB2 receptors in *Danio rerio* is involved in complex behaviors, including anxiety-related responses. Inhibition of the CB2 receptor activity in zebrafish leads to disorders in liver functions, which suggests the participation of the endocannabinoid system in lipid homeostasis [51]. Zebrafish have been shown to possess the *DAGLA* (diacylglycerol lipase α) homolog, with a quite similar pattern of expression to the one detected for CB1 receptors. The morpholino-induced transient knockdown of *DAGLA* homolog resulted in changes in axonal formation in the midbrain−hindbrain region, altered motion perception, and movement disturbances [48]. Scientists have also found *FAAH2* (fatty acid amide hydrolase 2) homolog in zebrafish, which is absent in rodents, but it is a component of the endocannabinoid system in humans [52].

## 3. Use of Zebrafish in Cannabinoid Research

### 3.1. Research on the Impact of Early Life Exposure to Cannabinoids

It has been demonstrated that both Δ^9^-THC and CBD cross the placenta, and they are present in breast milk [53]. Such an observation is highly important in view of the fact that up to 7.5% of pregnant women aged 18–25 smoke marijuana for recreational use [54]. At the same time, in their review based on epidemiological studies, Lagae [55] has drawn attention to the negative impact of cannabinoids on fetal life. Reduced birth weight, microcephaly, slow psychomotor development, and in the longer term—problems in school-aged children, including memory disorders or hyperactivity and aggression were listed by the author. Unfortunately, human observational studies on the impact of early life exposure to cannabinoids are limited due to many factors, e.g., small sample size, inconsistency in tested doses and exposure times, the variable composition of the smoked marijuana, as well as frequent co-use of other stimulants by pregnant women. Thus, it is impossible to clearly distinguish the specific effects of *Cannabis* on the fetus. As a consequence, on the one hand, researchers have been trying to improve observational studies [56], and, on the other hand, they are trying to use animal models to better understand the molecular mechanism of cannabinoid effects.

Zebrafish are a well-recognized animal model for studying early life stages and the developmental process because of the rapid development of *Danio rerio* embryos and larvae and because of the transparency of their body during early life [57]. Experiments in zebrafish embryos/larvae enable us to assess the toxicity of both well-known and newly synthesized compounds (including cannabinoids). Determination of LC50 (lethal concentration, 50%) or LOAEL (lowest observable adverse effects level) values makes it possible to compare the toxicity of different substances and define safe doses for further studies in adult animals. Developmental studies usually involve a continuous (chronic) exposure of *Danio rerio* from the gastrulation phase to a solution of a given tested substance and a simultaneous observation of the embryo/larvae up to 96 hpf. The following parameters are recorded: mortality rate, regularity of development, the occurrence of malformations including, for example, edema, disorders in the development of an ear or eye, pigmentation, body curvature, and hatching. Another type of toxicological experiment focuses on acute toxicity, in which zebrafish embryos are usually exposed to a given substance for 4–5 h during the gastrulation phase. Table 3 presents the main findings of the studies carried out within the last 5 years, investigating the effects of phytocannabinoids, hemp extracts, and synthetic cannabinoids on the early life stages in zebrafish. Since, as mentioned above, cannabinoids are quite frequently used with other drugs or ethanol, it is highly important to underline here that cannabinoids may interact with ethanol to perturb development. According to studies by Fish et al. [58], simultaneous exposure to Δ^9^-THC, HU-210 (a potent agonist of CB1 receptors), or CP-55940 (a potent and nonselective agonist of CB receptors) with alcohol induces a higher incidence of microphthalmia and midbrain/hindbrain boundary defects in *Danio rerio* embryos when compared to either treatment alone. Most probably, inhibition of the smoothened-dependent signaling by CB1 receptors was implicated in the development of the observed defects. The same results were obtained in a murine model, which confirms that outcomes obtained in zebrafish can be extrapolated to mammals. In experiments by Boa-Amponsem et al. [59], combined exposure to ACEA (a highly selective agonist of CB1 receptors) and ethanol induced microcephaly and microphthalmia.

### 3.2. Behavioural Research

Even though it is possible to carry out behavioral studies in the zebrafish model at an early stage of life (approx. 17 hpf), key areas in the brain of *Danio rerio* are homologous to the ones observed in mammals after 5 dpf. Thus, after 5 dpf, the pattern of zebrafish behavior in different laboratory tests can be compared to the activity of mice and rats. Behavioral tests in zebrafish are mainly used in the assessment of anxiety and stress, but they also enable the evaluation of the neurotoxicity of agents to which the larvae have been exposed. Well-known and well-described behavioral tests which are used in the zebrafish model are the novel tank test, open field test, and light/dark test. These behavioral paradigms closely resemble rodent tests that have been applied in experimental pharmacology for a long time. In behavioral tests, locomotor activity, swimming speed, traveled distance, and reactions of larvae to darkness and illumination are usually measured or observed [69,70]. The parameters mentioned above are correlated with anxiety behaviors in zebrafish, which makes the tests applicable in experimental projects focused on the activity of anxiolytic or antidepressant compounds [71]. Amongst cannabinoids, CBD is particularly considered a substance that could be useful in the management of psychiatric diseases, including anxiety disorders, which has been confirmed in both preclinical and clinical studies [72]. On the other hand, administration of Δ^9^-THC, particularly at high doses, can exert an anxiogenic effect and cause paranoia or psychosis [73], whereas its lower doses can improve anxiety symptoms [74,75]. According to Haller et al. [76], such a dual activity is associated with the fact that cannabinoids via CB1 receptors can affect GABA- and glutamatergic systems, which have an opposite effect on anxiety. Since CB1 receptors are located on both GABA and glutamatergic neurons, an interplay with the GABA neurotransmission may result in reduced anxiety, whereas interactions with glutamatergic mechanisms may lead to increased anxiety. Similarly, cannabinoids have the same CB1 receptor-dependent biphasic effect on novelty seeking and behavioral inhibition [77]. Furthermore, their dual activity toward appetite and body weight has been recently reported [78]. Therefore, research is needed to understand the effects of hemp and its respective compounds on behavior. Table 4 presents behavioral studies carried out within the last 5 years with the use of cannabinoids in the zebrafish model during the larval period and in adult subjects. Additionally, Boa-Amponsem and colleagues [59,79] demonstrated that per se ineffective doses of ACEA and ethanol when used in combination increased the risk-taking/anxiety-like behavior in juvenile zebrafish. The authors suggested that both substances have to share a common molecular mechanism of action, most probably, the inhibition of the Shh signaling and disturbances in the pathway involving Fgf8/Fgf19.

### 3.3. Drug Metabolism Studies in Zebrafish

Another important aspect related to the introduction of a new medicinal product into the pharmaceutical market is the assessment of the bioavailability and pharmacokinetics of its active substance. This is a crucial step during the research process, after which a large number of new molecules is usually rejected, and thus, they are not behaviorally stereotyped in further experiments.

The outcomes from animal studies obtained so far clearly indicate that the zebrafish model is suitable for the early stages of preclinical studies due to its similarity to rodents and humans in terms of the similarity of metabolic enzymes, proteins, and disease processes. Consequently, the above-mentioned similarities result in a correlation between the pharmacokinetics of a given substance in the human/rodent organism and its pharmacokinetics in zebrafish [88]. In the study by Park et al. [89], one of the synthetic cannabinoids (i.e., 4F-MDMB-BINACA) was administered via microinjection into the caudal vein, heart ventricle, and hindbrain in larvae 4 dpf. The mass spectrometry imaging revealed a high similarity between metabolites of 4F-MDMB-BINACA detected in zebrafish and metabolites of this compound detected in human biosamples. A 67% match rate was obtained when human blood samples were tested, and a 56% match rate was obtained when human urine samples were assessed. Morales-Noé and colleagues [90] investigated the metabolism of five new-generation synthetic cannabinoids (i.e., MMB-CHMICA, ADB-CHMICA, ADB-CHMINACA, MDMB-CHMCZCA, and NNL-3) in five-day-old *Danio rerio* larvae. The authors identified the metabolites of the tested compounds and determined their potential metabolic pathways [90]. The studies by Morales-Noé et al. [90] are also highly important because novel synthetic cannabinoids are used more and more frequently as drugs of abuse, and neither their metabolic pathways nor their effects are known. Up to 30% of all newly reported psychoactive substances are new-generation cannabinoids [91]. The use of the zebrafish model in metabolism studies may result in faster detection of cannabinoid metabolites in human samples.

### 3.4. Disease Modeling and Other Studies in Danio rerio

The similarity between the *Danio rerio* genome and the human one is 70%, which makes the zebrafish model suitable for studying diseases. According to the literature data [40], zebrafish have about 84% of genes that are known to be associated with diseases in people. Researchers have readily used transgenic or genetically modified *Danio rerio* lines to investigate diseases that occur in people. Outcomes of such experiments have confirmed a high level of similarity between the response to a given active substance in zebrafish and the response to the introduced treatment with the same active substance in humans. Methods and possibilities of disease modeling in *Danio rerio* were described in detail in the review by Patton et al. [88]. Accordingly, in the present paper, the main findings from studies related to the pharmacological activity of cannabinoids carried out in zebrafish disease models are summarized in Table 5. A great number of physicians and medical scientists have seen cannabinoids as a hope for new therapies in the management of diseases in which the treatment has been insufficient so far. One such health problem is drug-resistant pediatric epilepsy. Scientific evidence supporting the use of CBD in the treatment of epilepsy was discussed in the review by Arzimanoglou et al. [92]. The authors indicated that CBD had reduced the frequency of seizures by at least 50% in a significant proportion of patients participating in clinical studies. Furthermore, the authors summarized side effects related to therapy with CBD. The most frequently observed adverse reactions were gastrointestinal disorders, diarrhea, weight changes, changes in appetite, nausea, somnolence and fatigue, disorders of liver functions, pancreatitis, increased frequency of seizures, developmental regression, abnormal movements, and status epilepticus.

So far, only one medicinal product containing cannabinoids has been approved for the treatment of epilepsy by drug regulatory agencies in North America and Europe. It has already been mentioned in the Introduction section of the present review (in Table 2). At the same time, studies on newer and perhaps more effective compounds, including cannabinoids, have been carried out. Based on both outcomes of original papers [40] and data presented in reviews [93,94,95], the zebrafish model can be considered a well-described and broadly recognized laboratory animal species suitable for the assessment of an anti-epileptic activity of various agents. Parkinson’s disease, Alzheimer’s disease, or Huntington’s disease are other diseases in the management of which the use of cannabinoids could have a promising therapeutic value. Review articles indicate the neuroprotective effects of cannabinoids, which were demonstrated in rodents. Administration of these compounds prevented the loss of dopaminergic neurons, and it improved the motor functions of tested animals. Additionally, biochemical studies have shown their antioxidant, anti-inflammatory, and anti-apoptotic potential [96]. Another piece of evidence in favor of considering cannabinoid compounds in the treatment or prevention of neurodegenerative diseases is the role of the endocannabinoid system in the development of these diseases. CB receptors are present in brain structures responsible for planning and executing movements [97], and they participate in modulation of neuroinflammation in the microglia, as well as in regulation of the oxidative stress [98]. Parkinson’s disease can be studied in *Danio rerio* with the use of genetically modified lines (modulations related to *PARKIN*, *PINK1*, *DJ-1*, *SNCA*, *LRRK2* genes), animals with chemically induced symptoms (i.e., exposure to MPTP, 6-OHDA, rotenone, or paraquat), or subjected to chemogenetic ablation [99,100,101]. Apart from the abovementioned impact of the endocannabinoid system on the immune system, available data provide evidence that endocannabinoid signaling is important for cell growth, cell differentiation, cell metabolism, and apoptosis. Thus, both natural cannabinoids and their synthetic derivatives can be considered potential anticancer agents [102]. It has been demonstrated that agonists of CB receptors can have procancer activity, whereas antagonists of CB receptors exert anticancer effects [103]. Since mechanisms of the observed findings are complicated from the molecular point of view, there is a real need to investigate further the exact role of CB receptors in the development of cancers to understand the involved procancer and ant-cancer processes better and propose/introduce both novel and better therapeutic strategies. In fact, *Cannabis* extracts and cannabinoids are already used in the treatment of oncologic patients, and they are indicated for the alleviation of cancer pain and chemotherapy-induced nausea and vomiting. Based on data collected in Canada, where cannabinoids have been included in the clinical practice guideline related to the management of chronic pain [104], cancer patients report decreased intensity of pain, reduced opioid use, and improved quality of life [105]. Ellis and colleagues [106] evaluated the analgesic activity of several well-known painkillers (i.e., ibuprofen, tramadol, acetaminophen), Δ^9^-THC, and CBD in five-day old larvae that had been exposed to acetic acid. The authors confirmed that the applied animal model was suitable for the screening of new compounds for analgesic potential.

**Table 5:** Use of cannabinoids in the treatment of disease models induced in zebrafish.

Tested Substances	Tested Doses	Modeled Disease	Main Findings	Ref.
370 synthetic cannabinoid compounds	10 and 250 µM, and additionally, 1, 10, and 100 µM	Dravet Syndrome (*scn1lab* homozygous mutants, 5 dpf)	The used model successfully identified five synthetic cannabinoids with the anti-seizure potential, i.e., indole-based cannabinoids JWH 018 N-(5-chloropentyl) analog, JWH 018 N-(2-methylbutyl) isomer, 5-fluoro PB-22 5-hydroxyisoquinoline isomer, 5-fluoro ADBICA, and AB-FUBINACA 3-fluorobenzyl isomer.	[40]
CBD, cannabichromene (CBC), cannabidivarin (CBDV), cannabigerol (CBG), cannabinol (CBN)	CBD, CBN, and CBDV: 0.25–4 µM CBC: 0.1–3 µM CBG: 0.25–3 µM	Parkinson’s disease (6-hydroxydopamine (OHDA)-induced model, studies carried out 120 hpf)	The tested cannabinoids, when used separately, did not have any influence on the OHDA-induced hypoactivity, whereas when used as three-component equimolar mixtures (containing CBD + CBDV + CBC or CBD + CBN + CBC or CBD + CBDV + CBG) cannabinoids significantly reduced OHDA-related motor symptoms.	[107]
Crude CBD extract	0.1, 0.25, 0.5, 1.25 mg/L	Zebrafish embryo caudal fin amputation model (caudal fin was amputated 3 dpf, and observations were made 48- and 72-h postamputation)	CBD accelerated zebrafish fin regeneration in a dose-dependent manner, partially due to the regulation of the inflammatory response. It also reduced apoptosis after amputation.	[108]
CBD	1.25 µM	Tuberous Sclerosis Complex (tsc2 nonsense mutation, 6 dpf)	CBD exerted an anxiolytic effect in behavioral studies (anxiety closely associated with TSC). A decrease in the level of phosphorylated rps6 was observed, which may be due to a reduction in activation of TOR activation (TSC experimental models have increased mTOR activity).	[109]
THC, CBD	CBD: 1, 1.5, 2 µM THC: 1.5, 2, 3 µM	Neurohyperactivity (pentylenetetrazole-induced model and the genetic model induced by loss-of-function mutations in the GABA receptor subunit alpha1 (GABRA1−/−); 5 dpf)	Both THC and CBD opposed the behavioral hyperactivity induced by pentylenetetrazole and genetic mutation. Higher doses were needed in the case of the genetic model. THC and CBD acted synergistically when used concurrently.	[110]
Abnormal cannabidiol and O-1602	0.1–10 µM	Zebrafish xenograft model (taxol-resistant MDA-MB-231 cells of human breast cancer; 48 hpf	Both tested compounds induced apoptosis of breast cancer cells and they reduced cell migration at a concentration of 2 µM.	[111]
CBD, THC, CBVD, CBN, linalool (LN)	CBD: 0.3, 0.6, 1.0 µM THC: 1.0, 4.0 µM CBVD: 0.3, 0.6, 1.0 µM CBN: 0.3, 0.6, 1.0 µM LN: 0.3, 0.6, 1.0, 4.0 µM	Dravet Syndrome (*scn1lab* homozygous mutants) pentylenetetrazole-induced seizures; 6 dpf following 24 h of exposure	In the Dravet Syndrome model, CBD (0.6 µM), THC (1 µM), CBN (0.6 and 1 µM), and LN (4 µM) significantly reduced the number of seizures, with CBN being the most effective. In the pentylenetetrazole-induced model, only CBD and THC were effective.	[112]

## 4. Conclusions

Cannabinoids are a broad group of compounds of both natural and synthetic origin. Current knowledge about the functioning of the endocannabinoid system in the human body has opened a door for novel and promising therapeutic options with the use of CB receptor ligands in diseases lacking effective or sufficient treatment. As discussed in the present paper, the medical world sees the potential of cannabinoids in the management of epilepsy, neurodegenerative diseases, cancers, cancer pain, nausea and vomiting associated with chemotherapy, and eating disorders. Additionally, studies evaluating the potential use of cannabinoids in migraines, glaucoma, disturbances in the immune system, anxiety, sleeping disorders, or skin problems have been carried out [113,114]. Since preclinical experimentation is multidirectional, a relatively cheap and easily accessible animal model that requires a small quantity of the tested agent and yields the outcomes quite fast is needed. *Danio rerio* seems to be suitable for this role. Zebrafish may provide an alternative option to rodent models that have been used extensively in cannabinoid research [115,116]. *Danio rerio* have a well-described endocannabinoid system that bears a high similarity to the one in rodents and the human body. Zebrafish have similar CB receptors, endogenous ligands, and enzymes responsible for the biosynthesis and degradation of endocannabinoids as mammals. A 70% similarity was shown between CB1 proteins detected in zebrafish and humans/mice [18]. Outcomes of preclinical studies have confirmed that effects observed in zebrafish after exposure to cannabinoids (at least partially) correspond to the effects noticed in humans after administration of cannabinoids, including their teratogenicity, variety of behavioral responses, or even the specificity of cannabinoid metabolism. Furthermore, it is possible to induce a given disease in *Danio rerio* (e.g., Parkinson’s disease, epilepsy) that could be potentially treated with cannabinoids. Though literature data does not mention to which extent results from mammalian studies related to cannabinoids are similar to the results from zebrafish studies, there are reports confirming that the abovementioned genetic, physiological, metabolic, and pharmacologic conservation allows us to think of the zebrafish model as of a valuable tool to search for new drug targets, discover novel drugs, and model human disorders. In fact, a number of compounds that were discovered thanks to screening studies in *Danio rerio* have successfully entered clinical trials, which supports the zebrafish model as a translatable model system [88,117].

In conclusion, the collected data allow us to regard the zebrafish model as a suitable one for the preliminary phases of the cannabinoids research. This model complies with ethical regulations and it is recognized as a reasonable alternative to rodents when screening cannabinoid compounds for their toxicity profile and pharmacological properties.

## Figures and Tables

**Table 2:** Medicinal products containing cannabinoids approved by the FDA and/or EMA.

Drug	Indication
Epidiolex (oral solution containing 100 mg/mL of cannabidiol)	Adjunctive therapy of seizures associated with Lennox-Gastaut syndrome (LGS) or Dravet syndrome (DS), in conjunction with clobazam, for patients 2 years of age and older Adjunctive therapy of seizures associated with tuberous sclerosis complex (TSC) for patients 2 years of age and older
Marinol (capsules containing 2.5, 5, or 10 mg of dronabinol)	Anorexia associated with weight loss in patients with AIDS Nausea and vomiting associated with cancer chemotherapy in patients who have failed to respond adequately to conventional antiemetic treatments
Syndros (oral solution containing 5 mg/mL of dronabinol)	
Sativex (oromucosal spray containing 2.7 mg/mL of Δ^9^-tetrahydrocannabinol and 2.5 mg/mL of cannabidiol from *Cannabis sativa* L.)	Treatment for symptom improvement in adult patients with moderate to severe spasticity due to multiple sclerosis (MS) who have not responded adequately to other anti-spasticity medication and who demonstrate clinically significant improvement in spasticity-related symptoms during an initial trial of therapy
Cesamet (capsules containing 1 mg of nabilone)	Treatment of nausea and vomiting associated with cancer chemotherapy in patients who have failed to respond adequately to conventional antiemetic treatments

**Table 3:** Outcomes of studies investigating effects of cannabinoids on the early life stages in zebrafish.

Tested Substances	Tested Doses	Main Findings	Ref.
THC, CBD	THC: 2, 4, 6, 8, 10 mg/L CBD: 1, 2, 3, 4 mg/L	Acute experiments. A 5-h exposure during the gastrulation phase led to significant alterations in the heart rate, the morphology of motor neurons, synaptic activity at the neuromuscular junction, as well as changes in the locomotor responses to sound, as well as changes in the expression of postsynaptic nicotinic acetylcholine receptors.	[60]
THC	6 mg/L	Acute experiments. Embryos exposed to THC during the gastrulation phase presented minor changes in the axon diameter of Mauthner cells and minor changes in the escape response dynamic to touch. Furthermore, when developed until 5 dpf, animals presented reduced locomotor activity in comparison to the control group.	[61]
THC, CBD	THC: 1, 2, 4, 8, 16 µM CBD: 0.25, 0.5, 1, 2, 4 µM	Chronic exposure. A 96-h exposure to THC and CBD led to the development of oedemas, body curvature, and disorders in the formation of ears, eyes, and jaws. Animals presented behavioral changes. The LC50 for CBD and THC was 0.53 mg/L and 3.65 mg/L, respectively.	[62]
*Cannabis sativa* extract	2, 20, 200 µL	Chronic experiments. No negative effects on embryo development, embryo hatching, or survival rate were detected after exposure to the *Cannabis* extract. Larvae exposed to the highest dose of the extract (i.e., containing 1.73 nM and 22.3 nM of THC and cannabidiol CBD, respectively) showed increased locomotion compared to control groups. Moreover, qRT-PCR analysis showed that the highest dose of the extract induced an overexpression of CB1 and CB2 receptors.	[22]
CBN	0.25, 0.75, 1, 1.125, 1.2 mg/L	Chronic experiments. A 96-h exposure led to the development of malformations in zebrafish larvae, which increased in a dose-dependent manner. CBN concentrations > 0.75 mg/L induced the development of pericardial edema, anomalies in the yolk sac, tail bending, elongated hearts and shorter in their width, and separation of the ventricle from the atrium. The LD50 value was estimated at 1.12 mg/L. CBN acted as a stimulant and sedative agent, causing both altered velocity and bradycardia in animals.	[63]
THC	1, 1.25, 1.5, 2 mg/L	Chronic experiments (a 96-h exposure). The LC50 value was determined as 1.54 mg/L. Tail bending, pericardial edema, etc., were observed even in the LC50. The heart rate, cardiac contractility, and heart rhythm were also significantly affected.	[64]
5F-APINAC	0.001, 0.01, 0.1, 1, 10 µM	Acute and chronic experiments. An exposure to 5F-APINAC for 4 (acute treatment) and 96 h (chronic treatment) resulted in changes in GABA-, tryptamine-, acetylcholine-, xanthurenic acid-, picolinic acid-dependent neurotransmission. A 96-h exposure to the 5F-APINAC dose of 10 µM induced embryotoxicity (i.e., disturbed hatching process, tail bending, hyperpigmentation).	[65]
CBN	0.0.1, 0.1, 0.5, 1, 2, 3, 4 mg/L	Acute experiments (exposure from 5.25 to 10.75 hpf). Higher mortality rates, reduced mobility, and impaired development of motor neurons were detected with the increase in the tested doses of CBN. Even the lowest tested concentration induced disturbances in the development of hair cells responsible for the auditory function in zebrafish.	[66]
THC, CBD	THC: 0.024, 0.12, 0.6 mg/L; 0.08, 0.4, 2 µM CBD: 0.006, 0.03, 0.15 mg/L; 0.02, 0.1, 0.5 µM	Multigenerational experiments. After 96-h exposure of the F0 generation to THC and CBD no significant morphological abnormalities were detected in either F0 or F1 generations; however, signs of the developmental neurotoxicity were observed. Furthermore, the fecundity of the F0 generation was reduced, and after exposure to the highest tested dose of THC, animals from the F1 generation spent a significantly shorter time in the periphery.	[67]
THC, CBD	THC: 1, 2 µg/mL CBD: 1, 2 µg/mL THC + CBD: 1 + 1, 2 + 2 µg/mL	Acute experiments. Early exposure to CBD and THC during the first 10 h of embryonic development led to reduced neural activity in a dose-dependent manner when measured 4 days later.	[26]
CBD	0.02, 0.1, 0.5 µM	Multigenerational experiments. A 96-h exposure to CBD of the F0 generation resulted in increased survival and reduced body size in females. A dose of 0.5 µM reduced fertility in males, whereas a dose of 0.1 µM increased the production of eggs in females. These effects were not observed in the F1 generation.	[68]
CP-55940	1, 2.5, 3.8, 5.0 mg/L	Chronic experiments. A 24- or 48-h exposure to CP-55940 resulted in a dose-dependent increase in microphthalmia and midbrain/hindbrain boundary defects.	[58]
ACEA	1, 3, 6 mg/L	Chronic experiments. A 48-h exposure to ACEA at a dose of 6 mg/L induced microphthalmia in developing embryos.	[59]

**Table 4:** Behavioral studies with the use of cannabinoids in the zebrafish model.

Tested Substances	Tested Doses	Behavioral Test	Tested Subjects	Main Findings	Ref.
CBD	40 mg/L	Novel tank diving test	Adult fish (6 months)	A 30-min exposure to CDB decreased swimming speed and traveled distance.	[80]
CBD, WIN55,212-2	For both agents: 0.5, 1, 5, 10 µg/mL	Light/dark test	Larvae (4–5 dpf)	CBD at a dose of 10 µg/mL reduced the traveled distance and velocity of movement in the darkness. Both lower tested doses (0.5 and 1 µg/mL) of WIN55,212-2 reduced the traveled distance and moving duration, whereas higher doses of the agent (5 and 10 µg/mL) turned out to be lethal to zebrafish larvae within less than 24 h.	[81]
JWH-018	THC: 2 µM, JWH-018: 3 µM	Forced light/dark test, startle stimuli test, novel tank diving test	Larvae (5 dpf in the forced light/dark test; 6 dpf in the startle response), adult (4 months in the novel tank diving)	After the developmental exposure to THC and JWH-018, impaired locomotion was detected during baseline and dark periods in the forced light/dark test. In the startle stimuli test, no significant differences in the traveled distance before and during the stimuli were recorded for JWH-018, whereas reduced activity before the stimuli was detected for THC. In the novel tank diving test, adult zebrafish who had been subjected to developmental exposure to JWH-018 spent less time on the bottom of the tank, whereas developmental exposure to THC had no impact on animals’ behavior.	[82]
Whole-plant *Cannabis* extracts	0.25, 0.5, 1, 2 µg/mL	Light/dark test	Larvae (120 hpf)	Zebrafish larvae can be used to assess the bioactivity of *Cannabis* extracts. *Cannabis* extracts with various chemical profiles have a distinct impact on baseline larval activity and stress responses.	[83]
ACEA (CB1 receptor agonist)	1 mg/kg	Acute restraint stress, novel tank diving test	Adult	Treatment with ACEA prevented both the acute restraint stress-induced anxiety-like behavior and oxidative stress in the zebrafish brain.	[84]
THC	40 nM, 1, 2 µM	Swimming pattern	Adult	Animals exposed to THC presented abnormal swimming patterns, i.e., circular swimming (behavioral stereotypy).	[85]
THC, CBD, THC + CBD	THC: 0.05, 0.1, 0.5, 1.5, 2.0 µM CBD: 0.75, 1.0, 1.75, 2.5, 3.75 µM THC + CBD: 0.5 + 0.5, 1.5 + 0.5 µM	Locomotor activity in Zebrabox and the light/dark test	Larvae (120 hpf)	Exposure to THC (at all tested concentrations) decreased the locomotor activity of larvae, whereas exposure to CBD at concentrations above 1.75 µM increased locomotor activity.	[86]
9,10-dihydro-5-hydroxy-2, 3,6-trimethoxyphenanthrene-1,4-dione isolated from a commercial cannabis product	1, 2.5, 3.5 µM	Locomotor activity	Larvae (5 dpf)	Concentrations above 2.5 µM increased the locomotor activity of larvae	[87]

## Data Availability

No new data were created or analyzed in this study. Data sharing is not applicable to this article.
